# Escaping Host Immunity: New Tricks for Plant Pathogens

**DOI:** 10.1371/journal.ppat.1005631

**Published:** 2016-07-07

**Authors:** Ren Na, Mark Gijzen

**Affiliations:** 1 Department of Biology, University of Western Ontario, London, Ontario, Canada; 2 Agriculture and Agri-Food Canada, London, Ontario, Canada; The Sainsbury Laboratory, UNITED KINGDOM

## Introduction

Fungal and oomycete plant pathogens cause destructive diseases in crops and pose real economic and food security threats [1]. These filamentous, eukaryotic organisms can also upset natural ecosystems when they spread invasively [2]. The capability of plant immune systems to detect and respond to pathogen effector proteins is a major determinant of disease susceptibility. Plant pathogen effector proteins that trigger host immunity are often encoded by conditionally detrimental genes that are under strong and contrasting selective pressures [3,4]. Pathogen effectors evolved to play a positive role in virulence by enabling growth and reproduction on host plants [5,6]. Nonetheless, effectors can meet their match with host immune receptors that recognize their presence, a result that ends badly for the pathogen. Such immunity-triggering proteins are known as avirulence (Avr) effectors, encoded by *Avr* genes.

## Escaping Host Immunity

When an Avr effector triggers a host immune receptor, the pathogen’s survival depends upon generating variants that escape, suppress, or alter this recognition event in ways that allow the pathogen to grow and reproduce. This can be accomplished by numerous means. Transposon insertions or mutations to the DNA sequence encoding the *Avr* gene, or its complete loss, are commonly encountered gain of virulence mechanisms. This is well demonstrated in a study on the tomato leaf mold pathogen *Cladosporium fulvum* [7]. In fact, pathogen genomes have evolved configurations that position *Avr* effector genes in repetitive [8], transposon-rich [9], and teleomeric regions [10], or in dispensable segments [11], to aid mutation and recombination that results in gain of virulence. There are also ways to defeat immunity without loss or alteration of the DNA sequence of the offending *Avr* gene. This can occur through acquisition or evolution of an additional, epistatic effector that supresses the immune-triggering event caused by the Avr effector. Such scenarios are well documented in oomycete and fungal plant pathogens, for example, in the potato late blight pathogen *Phytophthora infestans* [12], the wheat powdery mildew pathogen *Blumeria graminis* [13], the tomato wilt pathogen *Fusarium oxysporum* [14], and the canola blackleg pathogen *Leptosphaeria maculans* [15]. This arms race can go through repeated iterations and lead to difficulties in untangling the molecular basis of host–pathogen compatibilities.

## Beyond Mutation

**Fig 1:**
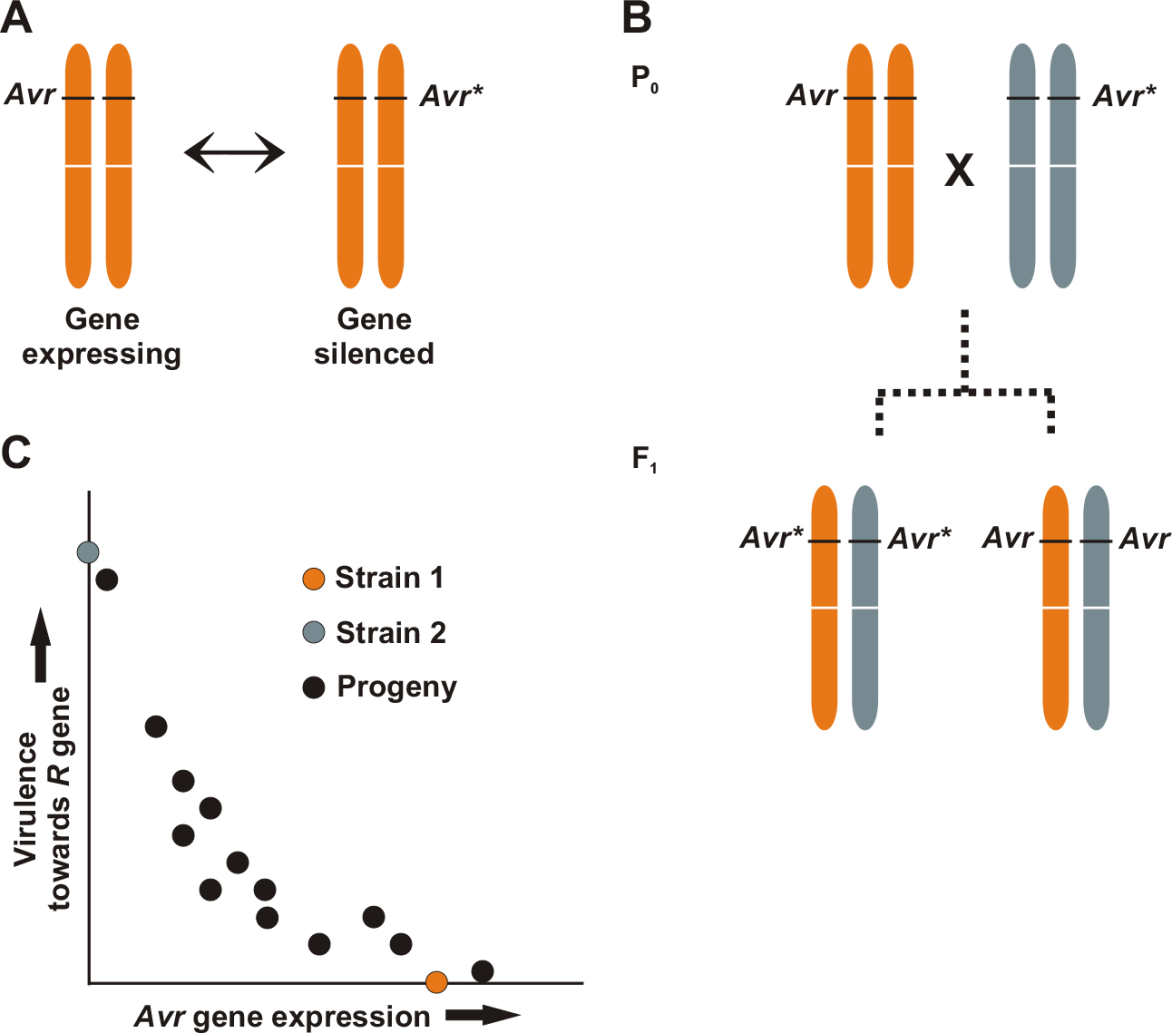
Avirulence (*Avr*) gene silencing in diploid oomycetes. **A,** Immunity-triggering effectors encoded by *Avr* genes can spontaneously switch between active (*Avr*) and gene silenced (Avr*) expression states in clonally propagating cultures. The *Avr* gene locus is depicted on a diploid chromosome pair. **B,** Sexual crosses between expressing (*Avr*) and gene silenced (Avr*) alleles can result in varying outcomes and unusual inheritance patterns for *Avr* gene expression in progeny. Progeny can differ qualitatively for *Avr* gene expression. Epigenetic reprogramming and strain-specific epistatic loci likely play a role in determining the result. The *Avr* gene locus is depicted on a diploid chromosome pair. **C,** Sexual crosses between expressing (*Avr*) and gene silenced (Avr*) alleles can also result in quantitative variation for *Avr* gene expression in progeny and incomplete penetrance of the avirulence trait when tested against host plants with the corresponding resistance (*R*) gene. The conceptual illustrations in this figure are based upon observations of *Avr1a*, *Avr1c*, and *Avr3a* expression and inheritance in the oomycete plant pathogen *Phytophthora sojae* [8,17,19,27].

Another way to escape immunity without changing the DNA sequence of the *Avr* gene is by altering its expression. This could simply result from shifting the DNA mutation to the *Avr* gene’s regulatory region or to an epistatic location in the genome that affects *Avr* gene transcription. Each of these mechanisms has been proposed to occur in the soybean root rot pathogen *Phytophthora sojae* [8,16]. Changes to *Avr* gene expression states that are not dependent on any DNA sequence alterations have also been postulated, based upon sequence-identical epialleles that differ in expression, as shown in Fig 1. The inheritance of *Avr* gene silencing in *P*. *sojae* shows unusual strain-specific patterns, including transgenerational effects [17], a phenomenon that is reminiscent of inter-nuclear silencing caused by ectopic expression of transgenes in *P*. *infestans* [18]. Although epigenetic inheritance of *Avr* gene expression states is controversial and difficult to prove conclusively, epigenetic switches that do not rely on DNA sequence changes offer the most plausible explanation for observations of reversible changes in virulence and *Avr* gene expression in clonally propagating cultures [19,20]. Virulence traits that are epigenetically reversible could impart a survival advantage to the pathogen by providing a means of recycling or re-deploying valuable effectors that are conditionally detrimental, in response to varying host immune capabilities [21,22]. Similar biological bet-hedging is observed in other successful pathogens. For example, knowledge of transcriptional control of antigenic variation in the malaria parasites (*Plasmodium* spp.) is comparatively far-advanced and provides another perspective on how pathogens recruit epigenetic systems that generate phenotypic variation in expression of immune-triggering effectors [23].

## A Big Role for Small RNA

Mechanistically, there is evidence that effector gene silencing in *P*. *sojae* and *P*. *infestans* is intertwined with small RNA pathways. Characterization of small RNA molecules in *Phytophthora* species show that the majority fall into two discrete size classes: approximately 21 nt and 25 nt [24,25]. The two sizes of small RNA differentially target transposons and effector gene families and associate with separate Argonaute proteins [26]. The *Avr3a* effector genes in *P*. *sojae* and *P*. *infestans* are non-orthologous, but each are subject to silencing that is naturally occurring and strain-specific [17,25]. In *P*. *sojae*, silencing of *PsAvr3a* has been linked to the presence of 25 nt small RNA. Genetic crosses between *PsAvr3a*-silenced and non-silenced strains can generate F_1_ hybrid progeny with unpredictable and variable levels of *PsAvr3a* gene expression. Hybrids display strain-specific effects that likely result from epigenetic reprogramming and from interplay of conventional and epigenetic variation between parental strains [17,27].

## The Challenges of Polyploidy

Gain of virulence mechanisms that rely on suppression of Avr triggered immunity or on epigenetic reprogramming of *Avr* gene transcription have the benefit of preserving the sequence of the *Avr* gene itself. For plant pathogens that are normally diploid or polyploid during their infective stages, such as the oomycetes, these mechanisms may additionally be favored because they can potentially offer an alternative to *Avr* gene dominance. Filamentous plant pathogens commonly exist in haploid, diploid, polyploid, or dikaryotic nuclear states during their host-infective stages. Sexual reproduction serves to re-assort alleles, but this could be limited by availability of complementary mating types for heterothallic species. A diploid, polyploid, or dikaryotic pathogen strain that possesses two or more functional copies of an *Avr* gene faces a different and arguably more difficult challenge to escape host immunity compared to a haploid organism possessing a single copy. This is because all copies of the *Avr* gene require gain of virulence changes. For example, consider a homozygous *Avr* gene in a diploid organism; a mutation to one allele results in heterozygosity for the *Avr* locus. This outcome does not achieve any phenotypic changes in virulence in the typical case of a dominant *Avr* gene. One way oomycete plant pathogens have evolved to compensate is through high frequency gene conversion or loss of heterozygosity [28–30]. Another adaptive solution is to regulate activity of the *Avr* gene through operationally dominant epistatic or epigenetic mechanisms or through stochastic switches. Studies of effector gene transcription in *P*. *sojae* and *P*. *infestans* offer evidence for these hypotheses [19,27,31,32]. Plant pathogens with dikaryotic infective stages, such as the cereal rusts, also have to counteract *Avr* gene dominance, so the oomycetes can be an instructive model in this instance. Certainly, it is intuitive that the ploidy status of the pathogen will influence the genetic-adaptive mechanisms that are employed to overcome host immunity. This could be especially pertinent in cases where gain of virulence relies on loss of function changes to effector genes that can either contribute to virulence or trigger host immune systems, in a conditional manner.

## Place Your Bets

The proposal that there are core sets of pathogen effectors that are indispensable and essential to virulence has become a popular idea that has pervaded molecular plant pathology [33–35]. Part of the attraction of this hypothesis is that it could offer opportunities for development of more durable plant resistance [1]. However, there is a need for perspective, because in host–pathogen interactions, outcomes are conditional. A precept of plant pathology is the concept of the disease triangle, which states that disease only occurs when host, pathogen, and environmental conditions are all permissive [36]. Extending this concept to effector biology is reasonable and would predict that the set of core effectors required for successful infection and reproduction is malleable and qualified by the host and environment. For effectors with the potential to trigger host immunity, selective forces drive redundancy and diversity, as exemplified by the RXLR (arginine-x-leucine-arginine) class of effectors in oomycetes [4,37]. Perhaps a useful analogy for this interplay between pathogen effector and host immunity components is a card game, in which some cards may have more face value than others, but ultimate success depends upon the combination of cards in hand and those held by opponents.

## Conclusion

The discovery of transcriptional and epigenetic variation of *Avr* gene expression in oomycetes provides an additional layer of knowledge as to how plant pathogens can escape host immune systems and adapt to changing selective pressures. The idea that epigenetic systems can enable effector recycling has been recently considered [21,22]. The hypothesis that epigenetic control of *Avr* gene expression is, in part, an adaptive response to *n* > 1 ploidy or nuclear states provides another rationale to explain the present set of observations. The prediction arising from this hypothesis is that epigenetic reprogramming of *Avr* gene expression will continue to be more frequently encountered as a gain of virulence mechanism in diploid, polyploid, and dikaryotic species compared to plant pathogens that are haploid during host infection.
